# The tailored sperm cell

**DOI:** 10.1007/s10265-017-0936-2

**Published:** 2017-03-29

**Authors:** Luis Alvarez

**Affiliations:** Center of Advanced European Studies and Research (caesar). Institute affiliated with the Max Planck Society, Ludwig-Erhard-Allee 2, 53175 Bonn, Germany

**Keywords:** Sperm morphology, Flagellar beat, Chemotaxis, Klinotaxis, Steering

## Abstract

Sperm are ubiquitous and yet unique. Genes involved in sexual reproduction are more divergent than most genes expressed in non-reproductive tissues. It has been argued that sperm have been altered during evolution more than any somatic cell. Profound variations are found at the level of morphology, motility, search strategy for the egg, and the underlying signalling mechanisms. Sperm evolutionary adaptation may have arisen from sperm competition (sperm from rival males compete within the female’s body to fertilize eggs), cryptic female choice (the female’s ability to choose among different stored sperm), social cues tuning sperm quality or from the site of fertilization (internal vs. external fertilization), to name a few. Unquestionably, sperm represent an invaluable source for the exploration of biological diversity at the level of signalling, motility, and evolution. Despite the richness in sperm variations, only a few model systems for signalling and motility have been studied in detail. Using fast kinetic techniques, electrophysiological recordings, and optogenetics, the molecular players and the sequence of signalling events of sperm from a few marine invertebrates, mammals, and fish are being elucidated. Furthermore, recent technological advances allow studying sperm motility with unprecedented precision; these studies provide new insights into flagellar motility and navigation in three dimensions (3D). The scope of this review is to highlight variations in motile sperm across species, and discuss the great promise that 3D imaging techniques offer into unravelling sperm mysteries.

## Introduction

Most species rely on sexual reproduction for the generation of new individuals. For this, male gametes (sperm) and female gametes (eggs) must be transferred from the specialized organs inside the body where they are produced to the fertilization site. Many different strategies have appeared during evolution to ensure the success of this step: Flowering plants, for instance, rely on the directed growth of pollen tubes that carry immotile sperm to the ovule of the female containing mature eggs (Dresselhaus et al. 2016); a process that relies on chemotaxis (Higashiyama et al. 2001). By contrast, in other plants and most animal species, sperm are endowed with the means to move and navigate to the egg by following physical and chemical signals. In the following, I outline the richness of sperm diversity.

## Sperm morphology and ultrastructure is diverse

**Fig. 1:**
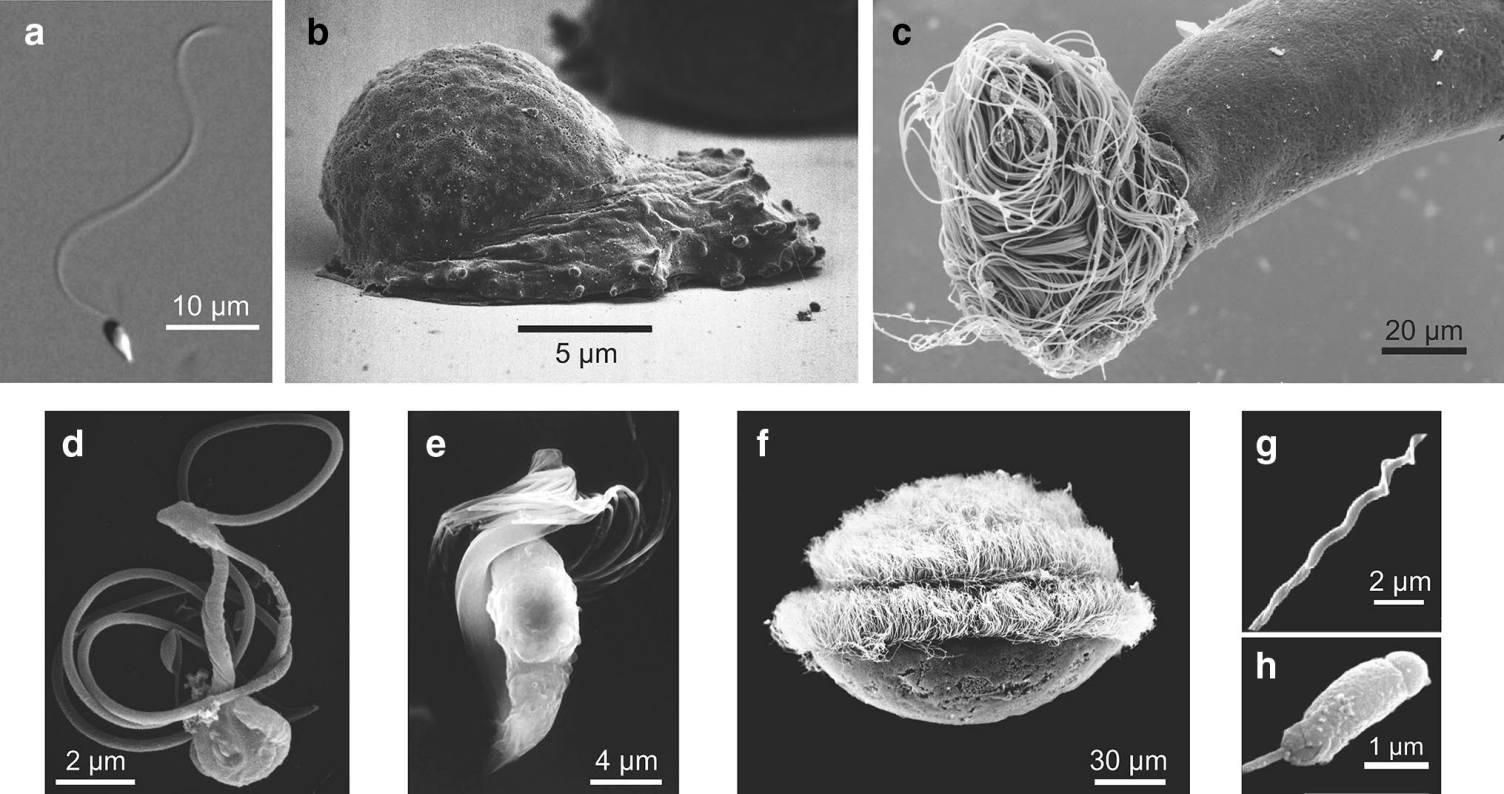
Exemplary variations in sperm design found in nature. **a** Sperm cell from the sea urchin *Arbacia punctulata*. The coarse overall morphology is found for many animal species. **b** The crawling sperm from the nematode *Ascaris suum* (picture courtesy of Dr. S. Sepsenwol). **c** The longest sperm known, from the fly *Drosophila bifurca* (picture courtesy of Dr. R. Dallai). Drosophila sperm with a shape similar to that of ball of wool is emanating from the last portion of the deferent duct. **d**–**f** sperm from different plants: *Conocephalum conicum* (d; biflagellated), *Equisetum hyemale* [**e**; with at least 80 flagella; (Renzaglia et al. 2002)], and *Cycas revoluta* (**f**; about 1000ths flagella). Reprinted with permission from (Renzaglia and Garbary 2001) and (Takaso et al. 2013). **g, h** Sperm can display profound variations in morphology even within the same class. Sperm from two passerine birds: the Eurasian bullfinch *Pyrrhula pyrrhula* (**g**) and the House sparrow *Passer domesticus* (**h**). Reprinted with permission from (Birkhead and Immler 2007)

**Fig. 2:**
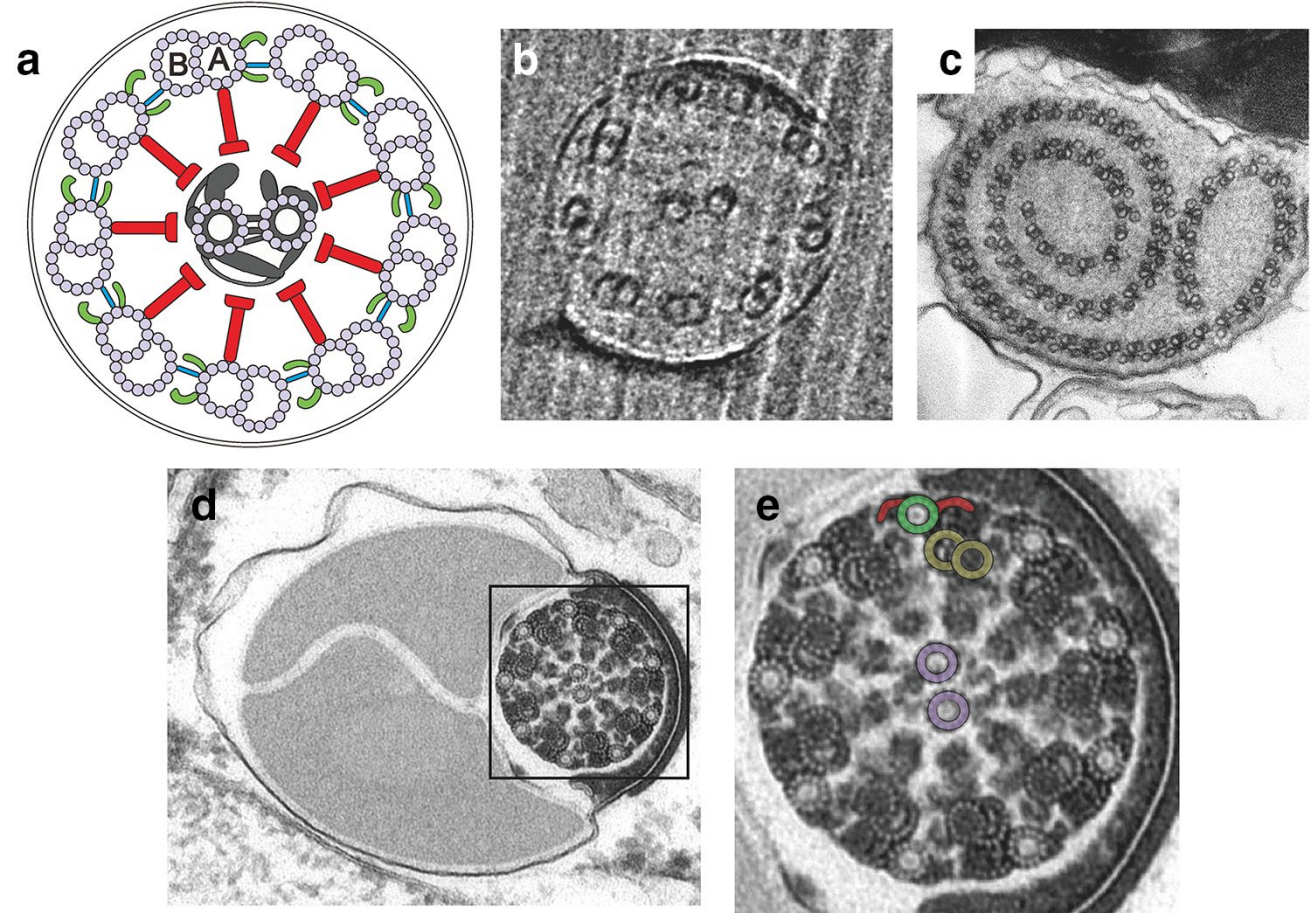
Exemplary axonemal structures found in sperm. **a** schematic diagram of a cross-section from a canonical 9 + 2 axonemal structure. View from the head towards the flagellar tip. Nine microtubule doublets (with subtubules A and B) are arranged cylindrically around an additional pair of microtubule singlets located at the centre. Two dynein arms (*green*) containing different subsets of dynein motors are attached to subtubule A and point towards the next B-tubule of the neighbour microtubule doublet in a clockwise direction (*green*). Dynein motors, fuelled by ATP, move adjacent microtubule doublets along the axis of the axoneme. Microtubule sliding is thought to be transformed into bending by mechanical constraints produced by their attachment to the basal body, nexin links (*blue*) (Lin et al. 2012), or clustering of motor activity (Movassagh et al. 2010). Radial spokes (*red*) connect the nine doublets in the periphery with the inner microtubule pair and associated filaments (*grey*). This complex is thought to orchestrate microtubule activity (Wargo and Smith 2003). **b** Flagellar cross-section from the sea urchin *Arbacia punctulata* with the canonical 9 + 2 structure. **c** Cross-section of the aberrant axoneme from the fly *Sciara coprophila* displaying its spiral shape. The axoneme is composed of 60–90 doublets, each one associated with a singlet or accessory microtubule. Subtubule A has two dynein arms (Jamieson et al. 1999). **d** Flagellar cross-section from the fly *Drosophila bifurca*. The axoneme and the two large mitochondrial derivatives can be seen. **e** Magnified detail from panel d displaying the characteristic 9 + 9 + 2 axoneme from insects. An accessory tubule (*green*) with its corresponding arms (*red*), a microtubule doublet (*yellow*), and the central pair (*violet*) have been labelled for clarity. Images are courtesy of Drs. S. Irsen (**b**) and R. Dallai (**c**–**e**)

The morphology of motile sperm varies drastically between species (Fig. 1). The structural motifs are so divergent, that sperm morphology has served to delineate the phylogenetic tree of mammals (Tourmente et al. 2011), insects (Dallai et al. 2016; Jamieson et al. 1999), and plants (Renzaglia and Garbary 2001). Nematode model organisms such as *Caenorhabditis elegans* and *Ascaris suum* produce sperm that move by crawling on a substrate similar to immune cells or the amoeba *Dictyostelium discoideum* (Fig. 1b) (Sepsenwol et al. 1989). The underlying cytoskeletal structures, however, do not rely on actin, tubulin or myosin, but on a conserved nematode- and sperm-specific protein (Batchelder et al. 2011; Sepsenwol et al. 1989). Most motile sperm cells use thin appendages named flagella or cilia for motility—in the following referred as flagella. Flagella share a highly conserved structural 9 + 2 motif (Mitchell 2007): a cylindrical arrangement of nine peripheral microtubule doublets crosslinked by dynein motors and a central pair of single microtubules (Fig. 2a) (Roberts et al. 2013). While motile sperm from most animals rely on a single flagellum at the rear of the cell for propulsion (Fig. 1a) (Cohen 1977; Jamieson et al. 1999), sperm from green algae and plants have multiple flagella. For example, sperm from *Charophycean* algae and bryophyte (mosses, hornworts, and liverworts) feature two flagella anchored at the head that extend backwards along the sperm body. Ferns and some gymnosperms, such as Ginkgo, have around dozens up to 1000 flagella. The sperm of *Zamia* (a cycad) can display up to 50,000 motile flagella (Renzaglia and Garbary 2001) (Fig. 1d–f). The length of a flagellum can also greatly vary from the short 1.7 µm of the termite *Reticulitermes lucifugus* to the 58 mm long sperm from *Drosophila bifurca* that is about 20 times the size of the male fly (Jamieson et al. 1999) (Fig. 1c). Part of this rich variation in sperm morphology has been attributed to the fertilization site. In particular, sperm from primitive external fertilizers display a rather homogeneous morphology (Cohen 1977). Internal fertilizers, instead, show marked differences in morphology, such as the head shape, even within closely related species (Birkhead and Immler 2007; Cohen 1977) (Fig. 1g, h). Sperm head design could result from sperm competition or interactions with the convoluted epithelium that lines the oviduct of the reproductive female tract. Finally, variations of the axonemal structure are also found that depart from the highly conserved 9 + 2 design (Fig. 2). From the 3 + 0 structure in the parasitic protozoan *Diplauxis hatti* to the giant axoneme of the dipteran *Asphondylia ruebsaameni* with 2,500 microtubule doublets (Mencarelli et al. 2001), variations are rich.

## Sperm competition and polymorphism

Due to sexual promiscuity in many species, successful fertilization requires sperm to outcompete rival sperm. This evolutionary pressure has driven many surprising aspects of sperm behaviour and morphology (Fitzpatrick and Lupold 2014; Lupold et al. 2016). Perhaps the best documented traits determining fertilization success are sperm swimming speed and numbers (Fitzpatrick and Lupold 2014; Gage and Barnard 1996). Yet, other traits such as sperm size, social cues, displacement of sperm from the stores in the female body (spermatheca), and many others exist in nature. Full description of these is beyond the scope of this review, for more detailed account see (Birkhead and Møller 1998). For sake of illustration, however, two examples are worth mentioning.

A perplexing case of sperm traits that originates from male competition is that of sperm trains (Foster and Pizzari 2010; Moore et al. 2002). In order to reach the egg first, sperm from rodents display a distinct behaviour by which hundreds or thousands of cells aggregate to form a train. These sperm trains swim at a speed that is about 50% higher than that of the individual cell. After 60–90 min, individual sperm cells dissociate from the train to make the last run for the egg. Another intriguing example of sperm traits that derive from male–male competition is that found in the squid *Loligo bleekeri* (Hirohashi et al. 2013; Iwata et al. 2011). In this species, two male types are found that exhibit different reproductive tactics. Large consort males compete with other males and court the female. If successful, these males will place their sperm capsules (spermatophores) inside the female (internal fertilizers). Small males (sneaker males) follow another tactic: these males will rush toward the female and dart their spermatophores into a seminal receptacle located out of the female body (external fertilizers). Sperm from consort and sneaker are morphologically different probably due to the different fertilization site (internal vs. external fertilization). Sperm from the large consort are smaller than that of the sneaker male (Iwata et al. 2011), but even more surprising, sneaker sperm display chemotaxis towards carbon dioxide and exhibit a swarming behaviour. The role of this chemotactic behaviour is unclear, but it has been speculated that CO_2_ might be used as a chemical signal to locate unfertilized eggs as they are extruded from the female (Hirohashi et al. 2013).

## Sperm signalling is divergent

For sensory cells such as photoreceptors, olfactory neurons and taste cells, signalling components are fairly well conserved. By contrast, the repertoire of proteins involved in sexual reproduction in general, and those making up sperm in particular, have been subject of extensive modifications during evolution (Birkhead and Immler 2007; Swanson and Vacquier 2002; Torgerson et al. 2002). As a result, signalling molecules controlling sperm motility are as diverse as sperm shape (Bönigk et al. 2009; Fechner et al. 2015; Kaupp and Strünker 2017; Strünker et al. 2015). The sperm-specific Ca^2+^ channel, CatSper, is an example that illustrates this signalling diversity. The CatSper channel complex is the most complex ion channel that has been identified so far. It features four pore-forming subunits and up to five auxiliary subunits, depending on the species. The channel probably appeared early in evolution before the branching of eukaryotes into the unikonts and bikonts (Cai et al. 2014; Chung et al. 2017). Although the control of CatSper by membrane potential and intracellular pH is conserved from sea urchins (Seifert et al. 2015) to mammals (Kirichok et al. 2006), the signalling mechanisms that eventually activate CatSper differ greatly. The female hormone progesterone, for instance, activates CatSper in humans (Lishko et al. 2011; Strünker et al. 2011), but not in mouse (Strünker et al. 2011). In human sperm, progesterone released by cumulus cells activates CatSper via an unconventional endocannabinoid mechanism (Miller et al. 2016); this progesterone-induced Ca^2+^ influx has been implicated in sperm chemotaxis (Oren-Benaroya et al. 2008; Publicover et al. 2008). Furthermore, chemicals as diverse as steroids, prostaglandins, odorants, and endocrine disrupting chemicals (EDCs) also activate human CatSper (Schiffer et al. 2014). It has been proposed that, for human sperm, CatSper serves as polymodal ‘stimulus integrator’, translating the chemical, hydrodynamic, and topographical microenvironment of the genital tract into a spatio-temporal pattern of Ca^2+^ signals (Brenker et al. 2012). In sea urchin sperm, CatSper activation involves a different signalling pathway initiated by the binding of a chemoattractant molecule to a chemoreceptor guanylate cyclase followed by the activation of two different ion channels and a Na^+^/H^+^ exchanger (Kaupp and Alvarez 2016). These signalling events produce changes in membrane potential and intracellular pH that are required for CatSper opening. Finally, even though sperm motility from the zebrafish *Danio rerio* is controlled by Ca^2+^ (Fechner et al. 2015), these species lack the genes for the CatSper channel (Cai and Clapham 2008).

Although Ca^2+^ signalling is ubiquitous in animals and plants, phylogenetic studies argue that the kit of Ca^2+^ channels and exchangers found in the different kingdoms differs extensively (Cai et al. 2014; Nagata et al. 2004; Wheeler and Brownlee 2008). For example, some land plants, such as *Arabidopsis thaliana*, are lacking the CatSper channel, four-domain voltage-dependent Ca^2+^ channels, and transient receptor potential channels. It would be interesting to enlarge the number of plant species in these studies to corroborate this finding. Interestingly, several cyclic nucleotide-gated channels (CNGC) have been identified in *Arabidopsis* (20) and the moss *Physcomitrella patens* (8), suggesting that this type of channels are abundant among plants. CNGC are key players in chemotaxis of sperm from sea urchins (Bönigk et al. 2009) and motility of Zebrafish (Fechner et al. 2015) and control Ca^2+^ entry in sperm. It would interesting to find which Ca^2+^ channels play a role in sperm chemotaxis among plants, and if CNGCs are involved.

In summary, signalling of sperm in most species involves changes in Ca^2+^, but the channels and the mechanisms of activation implicated differ greatly between species.

## The advent of 3D sperm imaging

Despite the fact that fertilization often takes place in three dimensions (3D), most of our knowledge on sperm motility and navigation is restricted to two dimensions (2D). The main reason for this lack of information is technological: When compared to crawling cells such as immune cells or *Dictyostelium*, most sperm move at speeds that are two to three orders of magnitude higher. Sea urchin sperm, for example, swim at speeds of about 200 µm/s (Jikeli et al. 2015). Dendritic cells or CD8^+^ T lymphocytes, by comparison, move at characteristic speeds of about 0.2 µm/s (Solanes et al. 2015). For this reason, tracking sperm in three dimensions (3D) with conventional imaging methods is challenging. Despite the technical difficulties, the 3D motion of sperm has been addressed for sea urchins and mammals, but very little is known for sperm from other animals such as insects or plants. Such lack of knowledge is striking, as insects represent by far the majority of species on our planet (Mora et al. 2011), and some of our current understanding of 3D sperm motility and navigation was first gathered from plants (Brokaw 1958). An influential and pioneering study of animal sperm motility in 3D was presented by Hugh Crenshaw (Crenshaw 1990). Using two dark-field microscopes with perpendicular views of an observation chamber, Crenshaw followed sea urchin sperm while swimming freely in 3D. This study provided experimental evidence for a mechanism of navigation called ‘helical klinotaxis’, by which microorganisms moving along a helical path align with a stimulus gradient by simply adjusting the cell rotation velocity as a function of the stimulus (Crenshaw 1996). After Crenshaw’s seminal contribution, imaging of sperm in 3D has not seen much progress until recently. Several studies have investigated sperm motility by following the 3D motion of the head (Corkidi et al. 2008; Di Caprio et al. 2014; Jikeli et al. 2015, Su et al. 2013, 2012) and the flagellum (Bukatin et al. 2015; Silva-Villalobos et al. 2014; Wilson et al. 2013), to list a few. In the following, I will discuss some insight provided by these studies into sperm motility, navigation, and steering using 3D methods.

### Sperm steering

A key aspect in flagellar motility concerns steering. Sperm propulsion involves a break of symmetry—the flagellar travelling wave moves in one preferential direction. For most animals, wave moves from the head to the tip of the flagellum. Sperm steering requires an additional symmetry break to change direction. Two mechanisms have been proposed to break this symmetry: an average flagellar curvature (Elgeti et al. 2010; Friedrich et al. 2010; Geyer et al. 2016) or a buckling instability (Bukatin et al. 2015; Gadêlha et al. 2010). In principle, both mechanisms involve bending of the flagellum into one preferential direction. The first mechanism assumes that bending is produced along the flagellum in a smooth manner. The second mechanism involves an abrupt bending that arises from strong mechanical flagellar compression along the axoneme. Bukatin and his co-workers used a novel method to examine the 3D flagellar beat of human sperm in a flow (Bukatin et al. 2015). Using common bright-field microscopy, the authors calculated the position of the flagellum along the axis normal to the focal plane from the broadening of the flagellum along its arc-length. This study describes several interesting aspects of human sperm motility: first, most human sperm produce a rolling motion along its longitudinal axis. Second, rolling is invariably counter-clockwise when observing the cell head-on towards the tip. Third, the cell population falls into two groups: those turning left and those turning right. Finally, turning direction correlates with bending of the flagellar midpiece: cells turning left feature an approximately zero average curvature, whereas cells turning right display a preferential flagellar bending to the right when observed from above. As pointed out by the authors, a preferential direction of bending cannot result from an average flagellar curvature, because due to rolling, the direction of bending should alternate from right to left. From this insight, the authors hypothesize that steering might be achieved by a dynamical buckling instability. While this study argues that a mean flagellar curvature is not sufficient to explain human sperm steering, the underlying mechanism remains to be shown experimentally. We expect new exciting surprises in this respect.

### Sperm are ambidextrous

**Fig. 3:**
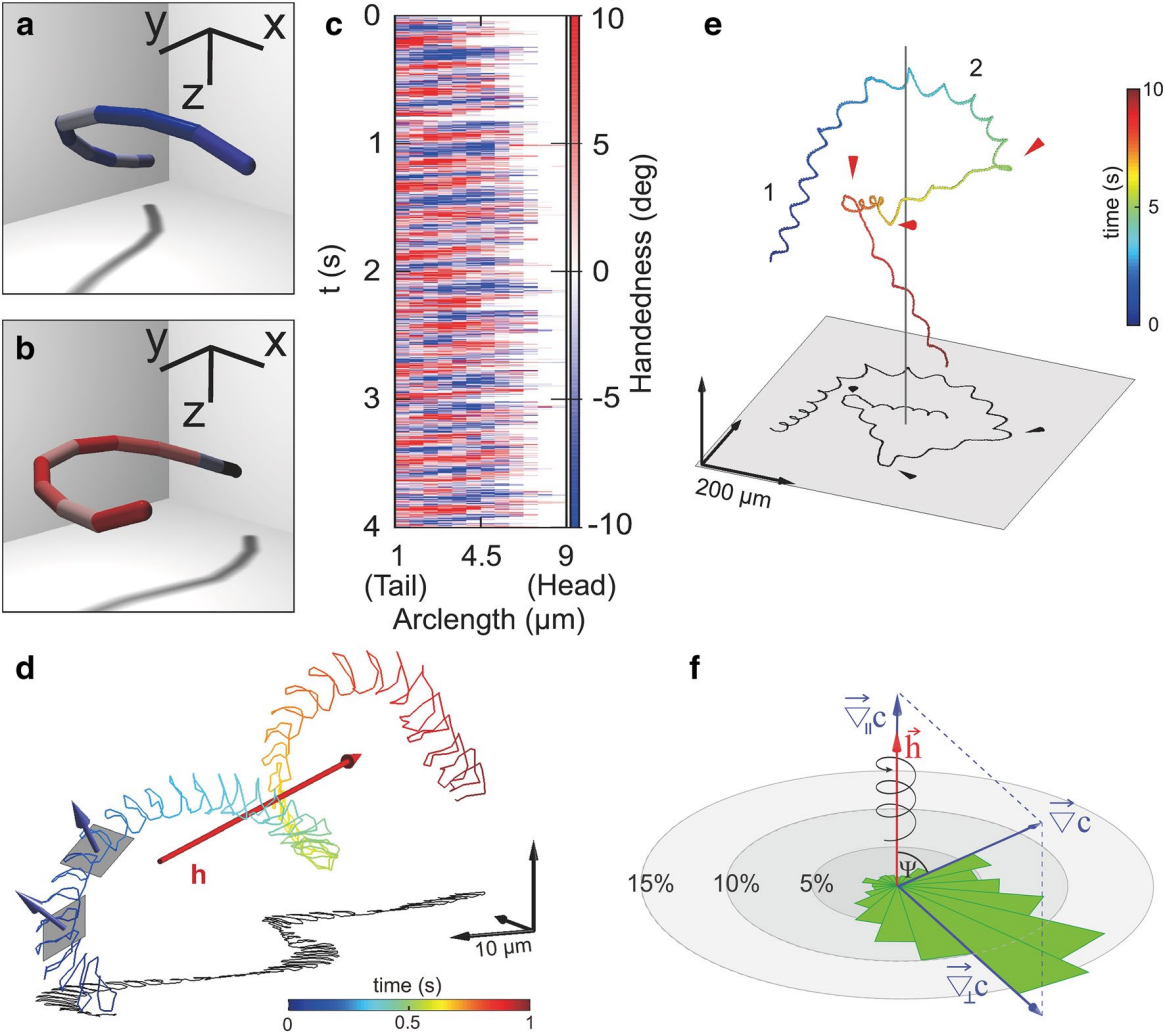
Recent advances in 3D sperm motility. **a**–**c** 3D reconstruction of the flagellum from malaria sperm. Flagella display a *left* (**a**) or *right* (**b**) handedness. The spatiotemporal map of chirality (**c**) shows propagating waves of alternate handedness across the flagellum. **d** 3D swimming path of a freely moving sperm cell from the sea urchin *A. punctulata*. Sperm swim along a helical path. From the motion of the sperm head, the flagellar beat plane can be inferred. Helix axis is shown in red. Two best-fitting planes along the path and the direction normal to them are shown in grey and blue, respectively. Time is color-coded along the path (see corresponding colorbar). **e** Swimming path of a sperm cell navigating in a chemical gradient. The axis of symmetry of the gradient is indicated by a *grey line* at the center. The cell approaches the gradient following a regular helical path (1). The helical axis bends smoothly during chemotaxis (2). Smooth helix alignment is interrupted by abrupt steering events (indicated by *red* cones). Strong alignment events occur when smooth alignment does not suffice to keep up-gradient swimming. **f** Rose plot of alignment events showing the changes in direction of the helical axis with respect to the parallel (${\nabla _{||}}c$) and perpendicular (${\nabla _ \bot }c$) gradient components. Alignment of the helical axis is not random but scatters around the perpendicular gradient component, showing that the helical axis bends deterministically to align with the gradient direction

Nodal cilia, found in embryos, represent an important exception to the 9 + 2 axonemal motif. These short appendages (about 5 µm long) are key to breaking the left–right symmetry of our body during embryogenesis and lack the central microtubule pair (9 + 0 motif). Their motion, in contrast to that of other motile cilia and flagella, is circular instead of roughly planar. This difference in beat planarity has been attributed to the absence of the central pair (Brokaw 2005; Hirokawa et al. 2009). However, other structures linking specific microtubule doublets might be involved as well (Lin et al. 2012). An essential feature of nodal ciliary beat is the sense of rotation. It has been observed that the circling motion is always in a single direction: clockwise - when observing these cilia from the cell base toward the tip (Hirokawa et al. 2009). This beat chirality is required for nodal cilia to break the left–right asymmetry formation during development of the embryo, and has been attributed to the underlying chirality of the axoneme itself: By design, dynein arms point in a clockwise direction from one doublet to the next (Fig. 2a) (Brokaw 2005; Hilfinger and Jülicher 2008; Hirokawa et al. 2009). Using holographic methods and sperm from malaria parasites as a simple flagellar model system to study sperm motility in 3D, Laurence Wilson revealed that, despite the intrinsic flagellar structural chirality, malaria sperm produce ambidextrous flagellar waveforms (Fig. 3a–c) (Wilson et al. 2013), i.e. the flagellar beat is left (Fig. 3a) or right handed (Fig. 3b), and handedness alternates at the frequency of the beat (Fig. 3c). This study represents a primary example on how knowledge about the mechanisms of action of the axoneme can be inferred for observations of the flagellar beat in 3D. As an aside, theoretical studies show that by tuning internal cellular parameters, such as structural elements or motor properties, a cilium could produce clock-wise and counter-clockwise beating. However, the probability of observing a specific turning direction is higher when the underlying structure is chiral (Brokaw 2005; Hilfinger and Jülicher 2008). Taken together, chiral rotation in nodal cilia and ambidextrous beating in sperm has been shown by experiments and predicted by theory.

### Sperm use a deterministic navigation strategy

**Fig. 4:**
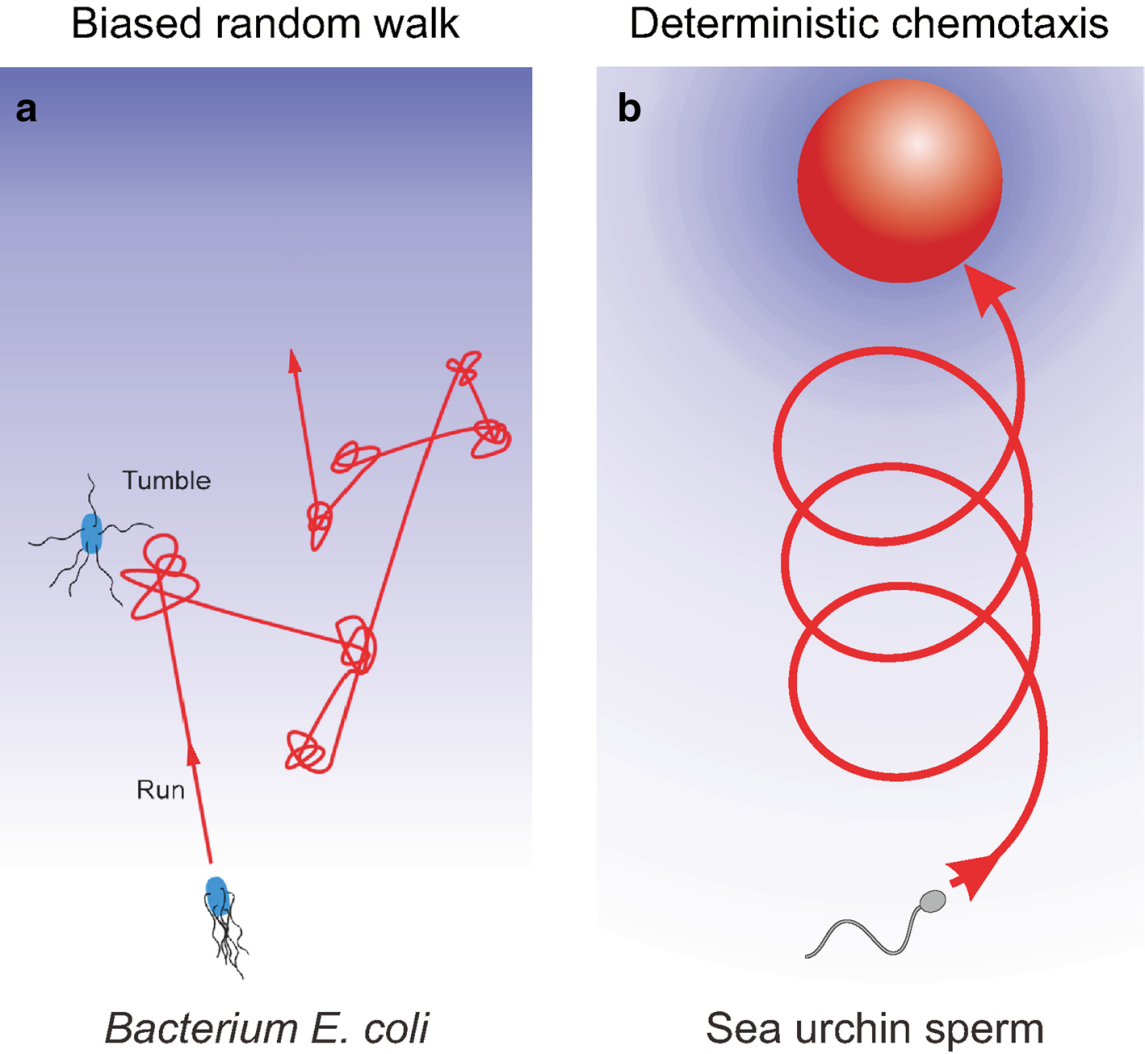
Sperm vs. bacterial navigation paradigm. **a**, Bacteria like *Escherichia coli*, use a navigation strategy that is adapted for small cells with high rotational diffusion that randomizes swimming direction. The strategy followed consists into alternation between straight swimming (runs), and stochastic changes in direction (tumbles). For chemotaxis, the duration of runs is extended when swimming up the chemical gradient. The interval between runs is about 1 s, which is shorter than the time at which cell orientation is fully randomized by rotational diffusion (Berg 1993). **b** Large cells like sperm are less prone to rotational diffusion and thus have a longer swimming persistence, which allows for a deterministic search strategy. At rest, sea urchin sperm swim along circles (near a wall) or helical paths (in 3D). Both swimming paths are characterized by a periodic component. When immersed in a chemical gradient, the periodic component of swimming results in periodic stimulation of the cell. Periodic stimulus is translated into deterministic steering by simply producing a periodic modulation of the path curvature (in 2 and 3D) and path torsion (in 3D) (Alvarez et al. 2014; Jikeli et al. 2015). Chemical gradient is shown in *shades* of *blue*

Many cells and organisms exploit physical and chemical cues to navigate in complex environments to search for mates, food or, more generally, better life conditions. Although crucially important, navigation principles along well-defined chemical gradients in three dimensions (3D) have been revealed only for bacteria. In his pioneering study (Berg and Brown 1972), Howard Berg revealed the stochastic navigation principle of bacterial chemotaxis that lead to the run-and-tumble model of chemotaxis (Fig. 4a) (Berg 1993). It took almost another half century of technological advances in several fields to address anew this complex and challenging question for a eukaryotic microswimmer. Using holographic microscopy, optochemical techniques, and computational models, Jan Jikeli et al. examined 3D navigation of sea urchin sperm while swimming in defined 3D gradients of a chemoattractant (Jikeli et al. 2015). The navigation principle in 3D is characterized by several key features. First, and consistent with previous reports (Corkidi et al. 2008; Crenshaw 1990), sperm swim along helical paths (Fig. 3d). Second, during chemotaxis, the helical swimming path bends in a deterministic fashion to align with the chemical gradient (Figs. 3e, f, 4b). Fourth, sperm respond to fast and slow components of the chemoattractant stimulus: A fast periodic component that results from the periodic component of helical swimming provides sperm with a local map of the gradient; it is used to steer in the right direction. Slow changes of the average stimulus level allow sperm to monitor its success and to trigger emergency steering responses in case sperm are veering off course (Fig. 3e). Finally, the mechanism of Ca^2+^ signalling underlying emergency steering responses, and the principle by which sperm detect increases and decreases of chemoattractant concentration were revealed, thereby, linking cellular signaling to cell behavior (Jikeli et al. 2015). Of note, emergency steering responses while swimming down the gradient have been also reported for sperm from algae, indicating that this cellular behaviour might be general (Kinoshita et al. 2017). The biflagellated sperm from these algae use their posterior flagellum for drastic turning. It would be interesting to know if the underlying signalling mechanism is conserved.

## Discussion

Sperm offer a unique example of evolutionary adaptation. These cells have been so extensively tailored that sperm from different species each merit their own studies. Variations in sperm morphology, ultrastructure, signalling, navigation principles, and behaviours are myriad. Our knowledge about sperm is vast, yet mostly compartmentalized, and does not cross interdisciplinary borders. For example, even though sperm chemotaxis has been described across most kingdoms (Yoshida et al. 2013), a comprehensive quantitative picture of sperm navigation and the underlying signalling mechanism has been advanced for only few model systems, such as marine invertebrates (Guerrero et al. 2010; Hirohashi et al. 2013; Kaupp and Alvarez 2016; Yoshida and Yoshida 2011; Zimmer and Riffell 2011), mammalian sperm (Bukatin et al. 2015; Eisenbach and Giojalas 2006; Kaupp and Strünker 2017; Miki and Clapham 2013), some fish (Fechner et al. 2015; Yanagimachi et al. 2013), and bracken fern (Brokaw 1958, 1973). For insects, most studies have addressed only morphological and evolutionary aspects. Sperm motility in insects has been only addressed sparsely and has remained for the most part descriptive. Despite the fact that some indications of sperm chemotaxis among insects have been reported (Grodner and Steffens 1978), no insect model for chemotaxis has been established. Males from some insects inject sperm trough a wound inflicted into the females abdomen (hypodermic insemination). These sperm must reach the females reproductive organs for fertilization (Jamieson et al. 1999). It would be interesting to study if chemotaxis plays a role into assisting sperm to find the spermatheca. In nematodes, it has been reported that sperm are able to perform chemotaxis (Sepsenwol CIL:44001). The role, mechanisms, and molecules involved are, however, still unknown. It is also perplexing, that although chemotaxis of plant sperm is firmly stablished, and these sperm cells are lacking a characteristic cell wall, fluorescent indicators have not been exploited to delineate the sequence of signalling events underlying chemotaxis in plants.

During the last decade, a number of studies using 3D techniques have tackled fundamental questions of sperm motility. Protozoa and insect sperm offer a unique opportunity to investigate axoneme architectures that depart from the prototypical 9 + 2 structural motif. Additionally, 3D imaging could be exploited to investigate sperm navigation in plants or algae. It would be interesting to see if mechanisms other than helical klinotaxis have evolved for these sperm species. Finally, sperm from most animals display a single flagellum that is used both for propulsion and as a rudder. Biflagellated sperm from algae (Kinoshita et al. 2017, 2016) and the liverwort *Marchantia polymorpha* (Miyamura et al. 2002) display flagellar waveforms that are different for each flagellum, and steering/propulsion can be adjusted differentially using both flagella. The beat of the posterior flagellum in *Marchantia* displays a more three-dimensional beat pattern than that of the anterior flagellum (Miyamura et al. 2002), indicating that possibly the posterior flagellum has an important role for 3D swimming. Algae sperm propel using both flagella, but steer by using the posterior flagellum as a rudder during chemotaxis (Kinoshita et al. 2017). The underlying signalling mechanism for differential beat patterns during chemotaxis of biflagellated sperm is unknown. More complex sperm, such as that of *Zamia* featuring up to 50,000 flagella could coordinate flagellar beating in a similar fashion as that found in the multicellular alga Volvox (Drescher et al. 2010; Ueki et al. 2010). It would be interesting to understand how multiflagellated plant sperm orchestrate 3D flagellar motion for directed motility.
